# Bridging large language model-based agents and workflow languages for automated multi-perspective analysis of single-cell RNA sequencing data

**DOI:** 10.1093/bioadv/vbag301

**Published:** 2026-10-08

**Authors:** Yoshinori Hayakawa, Haruka Ozaki

**Affiliations:** Laboratory for AI Biology, RIKEN Center for Biosystems Dynamics Research (BDR), Kobe, Hyogo 650-0047, Japan; Laboratory for AI Biology, RIKEN Center for Biosystems Dynamics Research (BDR), Kobe, Hyogo 650-0047, Japan

## Abstract

**Motivation:**

In single-cell RNA sequencing data analysis, the same dataset can be examined from multiple complementary perspectives, each requiring different analytical strategies and potentially leading to distinct biological interpretations. Systematically exploring such diverse strategies in a reproducible and scalable manner remains challenging.

**Results:**

We developed a framework combining large language model-based agents with workflow languages. Given an objective, the framework generates analytical strategies, converts each into a workflow plan, verifies that each plan reflects its intended strategy, executes accepted workflows in parallel, and integrates the outputs into a final report. Evaluation across three datasets and three large language models showed that plan validation improved the executable analysis intent fulfillment rate from 0.061 to 0.378. Diversity of analytical themes was established at as few as 10 generated strategies, while the number of distinct executable analysis paths grew continuously, reaching 59 unique paths at 100 generated strategies. Parallelization of both plan generation and workflow execution achieved up to a 29.9-fold speedup in workflow execution over sequential execution, with plan generation completing in near-constant time regardless of portfolio size.

**Availability and implementation:**

Source code is available at https://github.com/bioinfo-tsukuba/mpdas_paper under the MIT license.

## 1 Introduction

In exploratory data analysis, the choice of analytical strategy substantially influences the biological story that emerges from a given dataset. A single analysis pipeline cannot capture the full picture and risks overlooking important patterns and relationships (Yanai and Lercher 2020). Conversely, applying a diverse set of analytical strategies to the same dataset yields complementary perspectives, enabling more comprehensive biological insights. The importance of such multi-perspective exploration has been widely recognized across disciplines, including multiverse analysis in social science (Steegen *et al.* 2016) and highly comparative feature extraction for time-series data (Fulcher *et al.* 2013, Fulcher and Jones 2017).

This need is especially pronounced in single-cell RNA sequencing (scRNA-seq) data analysis. scRNA-seq quantifies gene expression at single-cell resolution (Gulati *et al.* 2025), and the analytical space is large, encompassing many branching points such as quality control, normalization, clustering, cell-type annotation, cell–cell interaction analysis, and pseudotime inference (Zappia and Theis 2021). Different analytical strategies can lead to different biological conclusions. Achieving multi-perspective scRNA-seq analysis therefore requires systematic, reproducible exploration of diverse analytical strategies.

Workflow languages such as Snakemake and Nextflow (Köster and Rahmann 2012, Di Tommaso *et al.* 2017) provide high reproducibility through dependency management and efficient resource use, but presuppose that analytical procedures are fixed in advance and are not designed for the flexible generation and exploration of diverse analytical strategies. In other words, they guarantee reproducibility but lack exploratory flexibility.

Large language model (LLM)-based agents (Xiao *et al.* 2026, Zhou *et al.* 2024, Alber *et al.* 2026, Chiu *et al*. 2026, Huang *et al.* 2026) address this flexibility constraint through code generation and tool execution. However, these systems are primarily designed to execute a single user-specified analytical strategy. In other words, existing LLM agents excel at flexible analysis execution but are not designed for systematic exploration of the analytical strategy space.

We propose a framework that combines the flexible planning capabilities of LLMs with the reproducible execution mechanisms of workflow languages (Fig. 1). We evaluate five aspects: (i) whether plan validation improves executable analysis intent fulfillment; (ii) whether the framework generates diverse strategies and analysis paths; (iii) whether large-scale exploration is practical through parallel execution; (iv) whether pre-execution planning offers concrete advantages over sequential LLM-agent execution in terms of robustness, speed, and portfolio-level execution management; and (v) whether integrated interpretive reports can be automatically generated.

**Figure 1:**
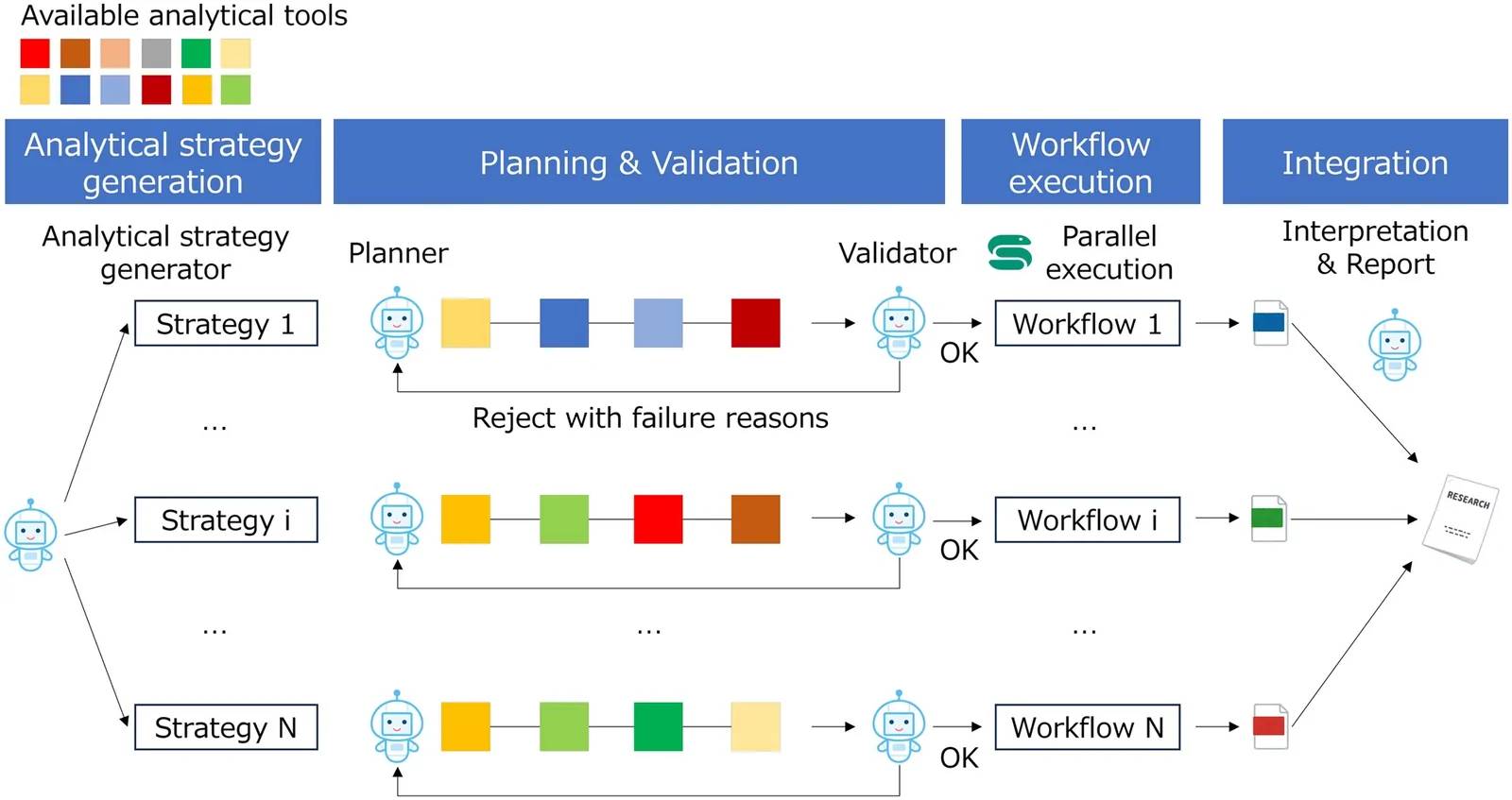
Overview of the proposed framework. From a research objective, the analytical strategy generator produces analytical strategies; each is converted into a Snakemake workflow via the planner and validator; validated workflows are executed in parallel; and the report generator integrates the outputs into an integrated interpretive report. The scRNA-seq analysis space is modularized as a tool catalog on MCP servers; colored squares represent individual tools exposed by each MCP server. Some illustrations in this figure were obtained from Irasutoya (https://www.irasutoya.com/) and used in accordance with its terms of use. These illustrations are not covered by the Creative Commons licence of this article. For permission to reuse them, please contact the copyright holder.

## 2 Methods

### 2.1 Framework overview

The framework consists of four steps (Fig. 1): (i) analytical strategy portfolio generation, (ii) workflow plan generation and validation, (iii) parallel workflow execution, and (iv) integrated report generation. The implementation uses Python 3.12 with uv for package management. The scRNA-seq analysis space is modularized as a tool catalog on model context protocol (MCP) servers, enabling LLM agents to construct workflows by selecting and chaining analysis steps.

### 2.2 Modularization of analysis steps

Analysis steps are redefined as computational modules with explicit input/output specifications, provided as endpoints of MCP HTTP servers. A total of 23 tools are available via 16 servers, covering QC and preprocessing, dimensionality reduction and clustering, cell-type annotation, cell–cell interaction, pseudotime analysis, transcription factor and pathway activity inference, signature scoring, gene module detection, cell fate mapping, gene set enrichment, gene dynamics, gene regulatory network inference, marker gene visualization, cell-type composition analysis, and gene expression program decomposition (Table 1). Each server is deployed as a Docker container and accessed via HTTP, eliminating per-workflow package installation overhead.

**Table 1:** Analysis tools available in the proposed framework.

Tool name	Function
qc_filter	QC filtering (Wolf *et al.* 2018)
normalize_and_hvgs	Normalization and highly variable gene selection (Wolf *et al.* 2018)
regress_and_scale	Regression and scaling (Wolf *et al.* 2018)
scrublet_doublet_detection	Doublet detection (Wolock *et al.* 2019)
cell_cycle_scoring	Cell cycle scoring (Wolf *et al.* 2018)
embed_and_cluster	Dimensionality reduction and clustering (Wolf *et al.* 2018)
find_markers	Marker gene identification (Wolf *et al.* 2018)
sctype_celltype_annotation	Cell-type annotation (Ianevski *et al.* 2022)
liana_cellcell_interaction	Cell–cell interaction analysis (Dimitrov *et al.* 2022)
paga_pseudotime_analysis	Pseudotime and trajectory analysis (Wolf *et al.* 2018)
decoupler_activity_inference	Transcription factor activity inference (Badia-I-Mompel *et al.* 2022)
decoupler_pathway_activity	Pathway activity inference (Badia-I-Mompel *et al.* 2022)
ucell_signature_scoring	Gene signature scoring (Andreatta and Carmona 2026)
scdrs_score	Disease-associated scoring (Zhang *et al.* 2022)
hotspot_gene_modules	Coexpression gene module detection (DeTomaso and Yosef 2021)
cellrank2_pseudotime_fate_mapping	Cell fate mapping (Weiler *et al.* 2024)
gseapy_ora	Over-representation analysis (Fang *et al.* 2023)
gseapy_gsea	Gene set enrichment analysis (Fang *et al.* 2023)
tradeseq_gene_dynamics	Gene dynamics along trajectories (GAM-based Python reimplementation inspired by tradeSeq; implemented via *pygam*)
inferelator_grn	Gene regulatory network inference (Skok Gibbs *et al.* 2022)
visualize_marker_genes	Marker gene visualization (Wolf *et al.* 2018)
celltype_composition_analysis	Cell-type composition analysis (Wolf *et al.* 2018)
run_cnmf	Gene expression program decomposition (cNMF) (Kotliar *et al.* 2019)

### 2.3 Analytical strategy generation

Given a research-objective metadata file and the MCP tool catalog, the analytical strategy generator produces the specified number of analytical strategies using batch generation and diversity-promoting prompts to suppress within-portfolio repetition. Each strategy is a natural-language sentence describing a specific analytical goal.

### 2.4 Dynamic plan generation and validation

For each analytical strategy, the planner selects and orders modules from the MCP catalog and represents the plan as a JSON-structured tool chain compiled into a Snakefile. The validator checks the plan along two dimensions: (i) *file-flow consistency*—a rule-based check that verifies each step’s required input is produced by a preceding step in the planned chain; and (ii) *strategy alignment*—an LLM-based check that judges whether the planned tool chain reflects the intent of the analytical strategy. If either check fails, the specific failure reasons are fed back to the planner to guide replanning; this loop is repeated up to a maximum of 20 iterations. Only accepted plans proceed to execution.

### 2.5 Workflow execution and report generation

Accepted Snakefiles are executed in parallel by Snakemake (Köster and Rahmann 2012). The MCP client distributes load across server replicas via hash-based routing, with replica count scaling with portfolio size.

Multiple workflow outputs are integrated by the workflow aggregator in three stages. First (generate-details), output files are interpreted by route: text/JSON by an LLM with research context, tables by an MCP server that safely executes LLM-generated Python code in an isolated environment, and images by a vision-language model (VLM). Second (generate-workflow-summary), per-file interpretations are aggregated into per-workflow summaries. Third (generate-final-report), all summaries are synthesized into an IMRAD-format integrated report.

### 2.6 Evaluation experiments

#### 2.6.1 Plan validation experiment

We evaluated the Validator across three datasets [peripheral blood mononuclear cells (PBMCs) (10x Genomics 2020), mouse brain tissue (10x Genomics 2024), Baron human pancreatic islets (Baron *et al.* 2016)] × 3 LLMs (GPT-4o gpt-4o-2024–08-06, GPT-5 gpt-5–2025-08–07, GPT-5-mini gpt-5-mini-2025–08-07), generating 20 analytical strategies per condition. Four metrics were assessed: (i) plan success rate, (ii) execution success rate, (iii) workflow addresses the intended analytical strategy, and (iv) executable analysis intent fulfillment rate (proportion of workflows that both executed successfully and satisfied the intended strategy, assessed by LLM-as-a-judge with GPT-4o). To assess the validity of the LLM-as-a-judge evaluation, an independent manual re-evaluation of all 360 cases was performed under blind conditions, without access to the LLM judge scores or reasoning, assessing only the alignment between the analytical strategy and the tool sequence.

#### 2.6.2 Diversity evaluation

Analytical strategies were embedded with sentence-transformers (all-MiniLM-L6-v2), visualized by uniform manifold approximation and projection (UMAP), and clustered into 10 themes by *K*-means (K=10). Structural diversity was quantified by the number of unique tool execution sequences across all Snakefiles.

#### 2.6.3 Scalability experiment

Portfolio sizes of 10, 20, 50, and 100 were tested on PBMC with GPT-4o. All workflows were launched simultaneously at maximum parallelism; sequential time was estimated from per-job wall-time sums.

#### 2.6.4 Comparison with ReAct

Three analytical strategies (S1: clustering and cell-type annotation; S2: cell–cell interaction analysis following cell-type annotation; S3: pseudotime analysis of monocyte lineage) were executed N=20 times per condition using the PBMC dataset and GPT-4o (gpt-4o-2024–08-06). A ReAct-based agent was implemented using LangGraph create_react_agent connected to the same MCP servers. Wall-clock execution time was measured for each strategy and condition; plan generation and Snakemake execution times for the proposed framework were extracted from run logs.

#### 2.6.5 Comparison with Biomni

The same 16 MCP servers used in the proposed framework were connected to Biomni (Huang *et al.* 2026) (GPT-5, gpt-5–2025-08–07) via its MCP interface. Two conditions were evaluated across N=3 independent runs on the PBMC dataset: (i) Biomni autonomously generated and executed 10 analytical strategies from research metadata alone and (ii) the same 10 fixed analytical strategies generated in the portfolio-size-10 experiment were provided directly. Task completion rate, output directory structure consistency across runs, and wall-clock execution time were recorded.

## 3 Results

### 3.1 Plan validation improves the executable analysis intent fulfillment rate

The purpose of the proposed framework is not merely to execute workflows, but to generate and execute workflows that fulfill the intent of the analytical strategy. We therefore conducted a systematic comparison with the Validator enabled and disabled across three datasets (PBMC, mouse brain tissue, and Baron human pancreatic islets) and three LLMs (GPT-4o, GPT-5, and GPT-5-mini), generating 20 analytical strategies per condition (Fig. 2).

**Figure 2:**
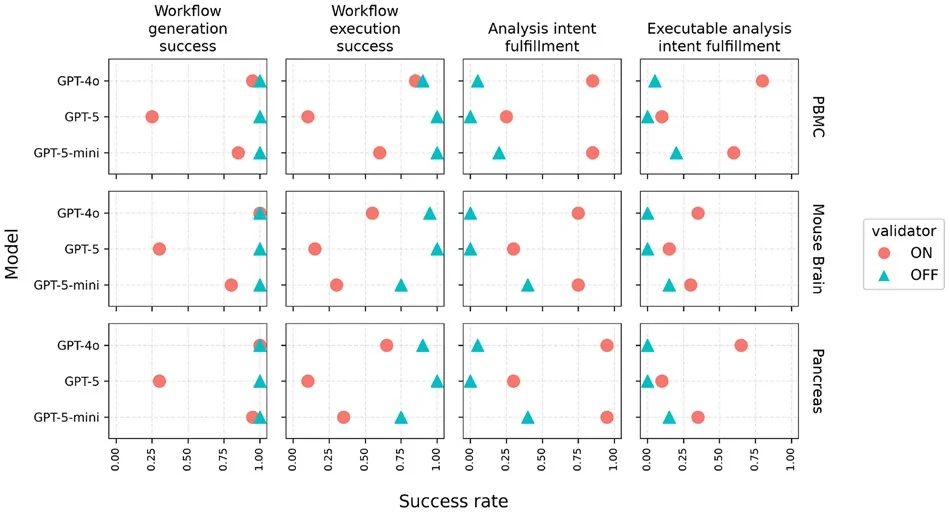
Comparison of conditions with and without the plan validation agent (validator) across 3 datasets (PBMC, Mouse Brain, Pancreas) × 3 LLMs (GPT-4o, GPT-5, GPT-5-mini), with 20 analytical strategies per condition. Metrics shown from left: workflow generation success, workflow execution success, analysis intent fulfillment, and executable analysis intent fulfillment. Red circles: with validation; blue triangles: without validation.

The most notable result is the rightmost metric, the executable analysis intent fulfillment rate: without validation the overall mean was 0.061, whereas with validation it improved to 0.378, consistently across all datasets and models. In contrast, plan success rates and execution success rates were in some cases higher without validation. This is because, without validation, technically executable but analytically incomplete workflows that do not adequately fulfill the intent of the analytical strategy are generated and executed. In other words, high execution success rates do not guarantee analytical validity, and plan validation is indispensable for generating meaningful workflows.

Improvement varied by model: GPT-4o showed the largest gain (mean across three datasets: 0.017→0.600), GPT-5-mini a moderate gain (0.167→0.417), and GPT-5 the smallest (0.000→0.117). The PBMC dataset yielded the highest fulfillment rate (mean across models: 0.500). These results indicate that the effectiveness of the validator depends on model capability, suggesting that LLM model selection is an important factor governing workflow planning quality.

The overall agreement between the human judge and the LLM judge was accuracy = 92.2% and Cohen’s κ = 0.836, indicating a high level of agreement (Landis and Koch 1977). In the GPT-4o (validator-enabled) condition, κ was low (0.040) reflecting the known prevalence effect of Cohen’s κ under highly imbalanced class distributions (human positive rate: 92%), but accuracy = 80.0% still indicates substantial agreement. These results are consistent with the validity of the LLM judge and mitigate concerns regarding potential self-reinforcement bias.

### 3.2 The framework generates diverse strategies and analysis paths

Because the purpose of this study is to explore the analytical strategy space itself, we evaluated whether the framework can generate diverse strategies and analysis paths at two levels, using the PBMC dataset and GPT-4o at portfolio sizes of 10, 20, 50, and 100.

#### 3.2.1 Diversity of analytical themes

Strategies were embedded with sentence-transformers (all-MiniLM-L6-v2) and clustered into 10 themes by *K*-means (K=10). Even with as few as 10 strategies, 5 of 10 analytical theme clusters were covered, including transcription factor activity analysis, signal pathway activity analysis, pseudotime analysis, gene regulatory network analysis, and gene expression program analysis (Fig. 3a and b). Coverage increased to 8 of 10 themes at 20 strategies and 9 of 10 at 50 strategies. Increasing portfolio size did not shift the distribution in UMAP space, demonstrating that thematic diversity is governed by the generator design rather than portfolio size.

**Figure 3:**
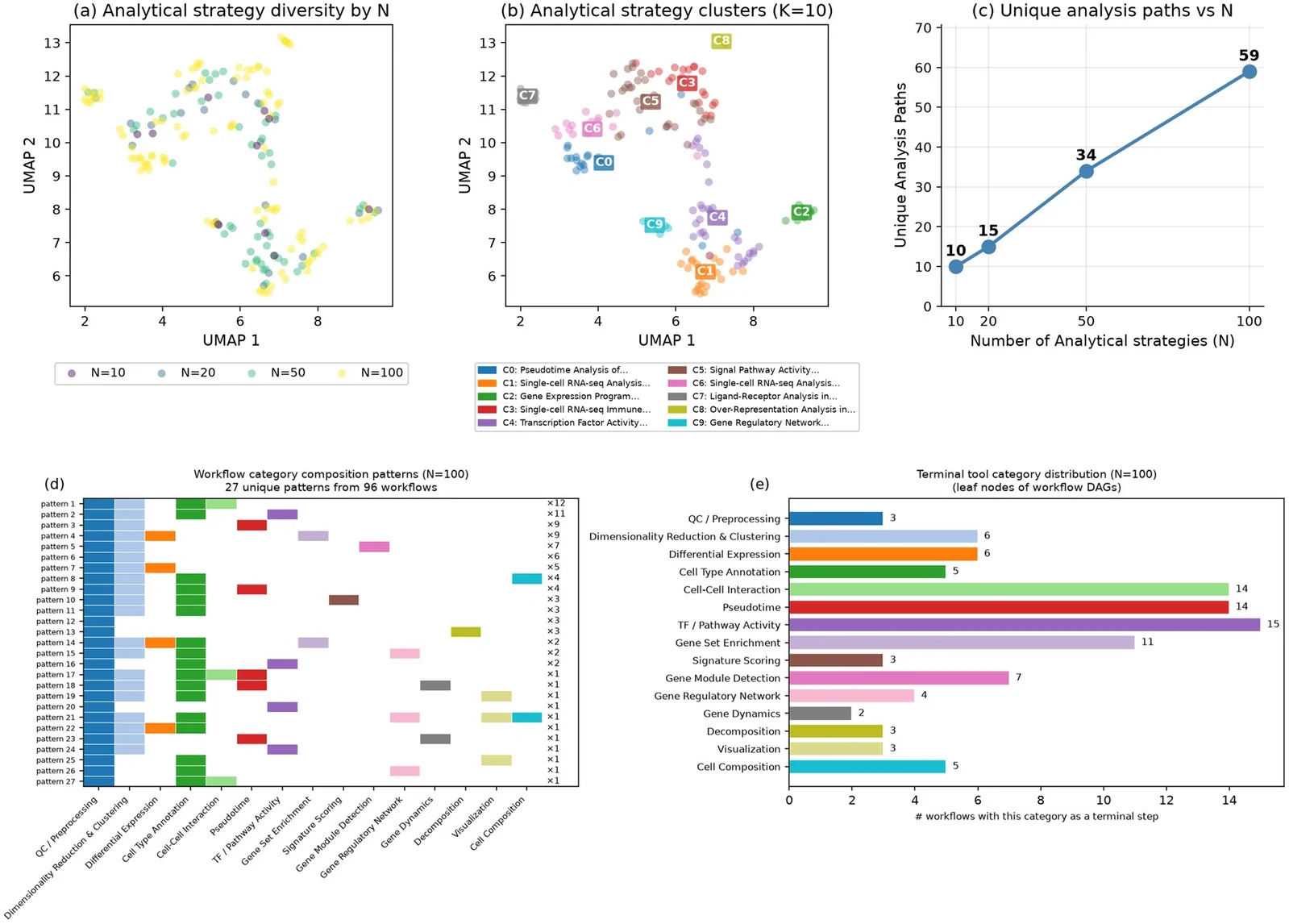
Diversity of analytical strategies across strategy set sizes (PBMC, GPT-4o). (a) UMAP projection of all analytical strategies (N=10–100), colored by strategy set size. (b) The same UMAP colored by *K*-means cluster (K=10; C0–C9). Each color represents a distinct analytical theme cluster. (c) Number of unique analysis paths as a function of strategy set size. (d) Category composition matrix of 96 workflows at portfolio size 100 (*N* = 100 PBMC experiment); each row is a unique category composition pattern, each column a biological analysis category, and ×n at the right indicates the number of workflows with each pattern. (e) Distribution of terminal tool categories across all workflows; numbers at the end of the bars indicate the number of workflows terminating in each category.

#### 3.2.2 Analysis path diversity

The number of unique tool execution sequences (analysis paths) across all Snakefiles increased monotonically with portfolio size, reaching 59 unique paths at portfolio size 100 with no sign of saturation (Fig. 3c).

#### 3.2.3 Diversity of biological analysis objectives

To assess whether distinct tool sequences address biologically different questions rather than converging on identical conclusions, we categorized each of the 23 MCP tools into one of 15 biological analysis categories and examined the category composition of each workflow. At portfolio size 10 (*N* = 10 PBMC experiment), 9 unique category composition patterns were observed across 10 workflows; at portfolio size 100, 27 unique patterns emerged from 96 valid workflows (Fig. 3d). The terminal categories—representing the final analytical objective of each workflow—spanned 13 distinct categories, with TF/pathway activity (15 workflows), cell–cell interaction (14), and pseudotime (14) as the most frequent (Fig. 3e). Because workflows terminating in cell–cell interaction (ligand–receptor signaling) and pseudotime (cell differentiation trajectories) address fundamentally different biological questions, convergence on identical conclusions is structurally unlikely even when applied to the same dataset.

In summary, the framework generates diversity at three levels: diverse analytical themes are maintained even with a small portfolio, the breadth of analysis paths executed expands further as portfolio size grows, and biological analysis objectives span structurally distinct categories.

### 3.3 Parallelization of plan generation and workflow execution makes large-scale exploration practical

To quantify the practical benefit of parallelizing both plan generation and workflow execution, we measured wall-clock time at portfolio sizes of N=10, 20, 50, and 100 using the PBMC dataset and GPT-4o (Fig. 4). Because each analytical strategy is planned independently, all *N* plans were generated simultaneously. For each phase, a sequential baseline was estimated: for plan generation, by multiplying the observed parallel planning time by *N* (a lower-bound estimate, as actual serial execution would additionally incur API rate-limiting overhead); for workflow execution, by summing the per-job wall-clock times recorded during the parallel run.

**Figure 4:**
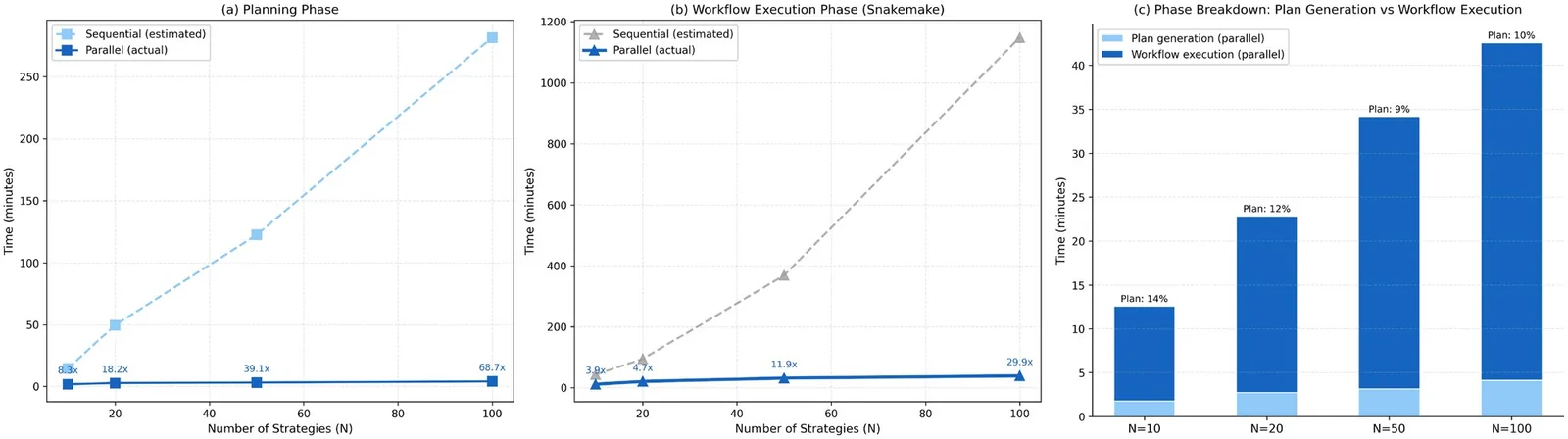
Scalability of plan generation and workflow execution phases (PBMC dataset, GPT-4o). (a) Wall-clock time as a function of strategy set size for the plan generation phase, comparing parallel execution (actual) against a sequential estimate. (b) Wall-clock time for the workflow execution phase, comparing parallel execution (actual) against a sequential estimate (sum of per-job wall-clock times). (c) Breakdown of total parallel wall-clock time into plan generation (light blue) and workflow execution (dark blue), with the percentage attributable to plan generation annotated above each bar.

Parallel plan generation completed in near-constant time across all tested sizes—105 s at N=10 and 246 s at N=100—whereas the sequential estimate scales linearly with *N*, reaching ∼281 min at N=100. This demonstrates that the plan generation phase does not become a bottleneck as the number of strategies grows (Fig. 4a).

For workflow execution, the sequential estimate increases from 2541 s at N=10 to 68 909 s ( ∼19 h) at N=100. Parallel execution completed the same workload in 649 s at N=10 and 2307 s ( ∼38 min) at N=100, achieving speedups of 3.9×, 4.7×, 11.9×, and 29.9× at N=10, 20, 50, and 100, respectively (Fig. 4b).

In the fully parallelized implementation, plan generation accounted for only 9%–14% of total wall time at all tested sizes, with workflow execution constituting the dominant phase (Fig. 4c). Because each analytical strategy is an independent workflow, both phases can be fully parallelized, enabling large-scale multistrategy exploration within a practical timeframe.

### 3.4 Comparison with sequential agent systems

#### 3.4.1 Pre-execution planning improves robustness and speed over ReAct

To examine the practical consequences of explicit workflow planning versus sequential LLM-based tool execution, we compared the proposed framework with a ReAct-based agent (Yao *et al.* 2023) that accesses the same MCP tool catalog across three analytical strategies (N=20 per condition, PBMC, GPT-4o; Fig. 5).

**Figure 5:**
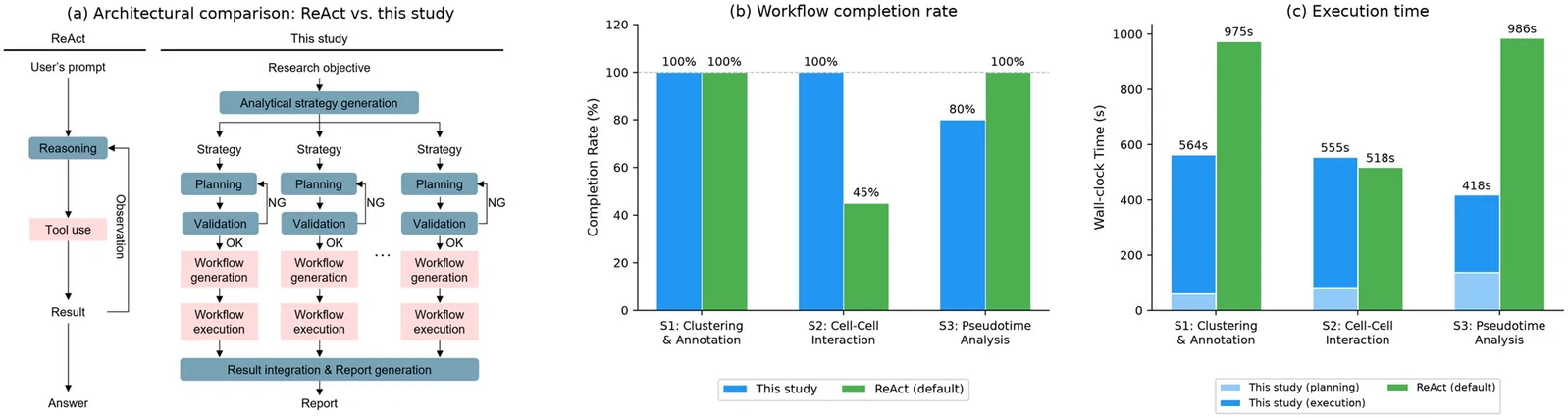
Comparison of the proposed framework and ReAct (Yao et al. 2023) across three analytical strategies (N=20 per condition, PBMC, GPT-4o). **(**a) Architectural comparison. (b) Workflow completion rates. (c) Wall-clock execution time (proposed framework bars are stacked: planning phase in light blue, Snakemake execution in blue).

The two approaches differ fundamentally in how tool-execution order is determined. In the proposed framework, a planner generates a complete workflow before any tool is called, and a validator checks file-flow consistency and strategy alignment before execution proceeds. In ReAct, tool-execution order is decided at each step by the LLM based on its current observation, without prior structural verification.

This difference in execution architecture had concrete consequences in S2 (cell–cell interaction analysis following cell-type annotation). Eleven of 20 ReAct runs invoked cell-type annotation directly on the raw input file, bypassing prerequisite preprocessing steps and terminating with an error. Critically, the remaining 9 of 20 runs with the identical strategy and model succeeded—demonstrating that this failure reflects intrinsic stochasticity of sequential LLM inference rather than a flaw in the strategy specification. The file-flow constraint enforced by the validator structurally prevents this failure pattern by construction.

Overall workflow completion rates were 93% (56/60) for the proposed framework and 82% (49/60) for ReAct (Fig. 5b). For S1 and S3, where both approaches completed all or nearly all runs, the proposed framework was approximately two-fold faster (S1 + S3 combined: 992 s versus 1961 s; Fig. 5c). This speed advantage arises from parallel execution of both plan generation and Snakemake workflows, whereas ReAct executes tools sequentially.

#### 3.4.2 Portfolio-level execution management is difficult to achieve with sequential agent execution

To assess whether a biomedical agent equipped with the same tool suite and based on sequential tool execution can replicate portfolio-level management, we connected the same 16 MCP servers to Biomni (Huang *et al.* 2026) and provided it with research metadata to generate and execute 10 analytical strategies (N=3 independent runs). Under both the condition in which Biomni autonomously generated analytical strategies and the condition in which fixed strategies were provided, nearly all strategies produced outputs across all three runs, confirming that MCP-connected Biomni is a capable baseline for executing individual scRNA-seq analyses.

However, three important differences remained. First, *auditability*: the proposed framework saves the execution plan for each workflow in a structured format, enabling immediate inspection of what was executed, in what order, and with what dependencies. Biomni produces no equivalent artefact, and tracing execution requires parsing raw agent logs—a challenge for both human reviewers and automated processing. Second, *output structure consistency*: because the proposed framework fixes output paths at the planning stage, the structure of produced artefacts is identical across runs. Because Biomni generates code from scratch at each run, the directory structure of produced artefacts varied across runs, complicating downstream processing including integrated interpretive report generation across the full portfolio. Third, *scalability*: Biomni executed all analyses sequentially across all runs, requiring 38–44 min per run at N=10. The proposed framework completed the same N=10 workload in 13 min via parallel execution, and achieved ∼30-fold speedup over sequential execution at N=100. These differences reflect the use of pre-execution planning rather than differences in model capability, and are therefore unlikely to disappear solely through improvements in the underlying LLMs.

### 3.5 Integrated reports translate multiperspective outputs into interpretable knowledge

To demonstrate that multiple workflow outputs can be integrated into interpretable knowledge, we applied the workflow aggregator to the PBMC portfolio-size-10 results. The outputs of 10 workflows—including UMAP visualizations, marker gene tables, pseudotime trajectories, and cell–cell interaction networks—were collected, and the report generator produced an IMRAD-format integrated report (Fig. 6). The generated report integrates analytical perspectives spanning cell-type identification, pseudotime trajectories, cell–cell interactions, and gene regulatory networks into a single document, demonstrating that executing a portfolio of analytical strategies leads to an integrated description of multi-perspective biological insights.

**Figure 6:**
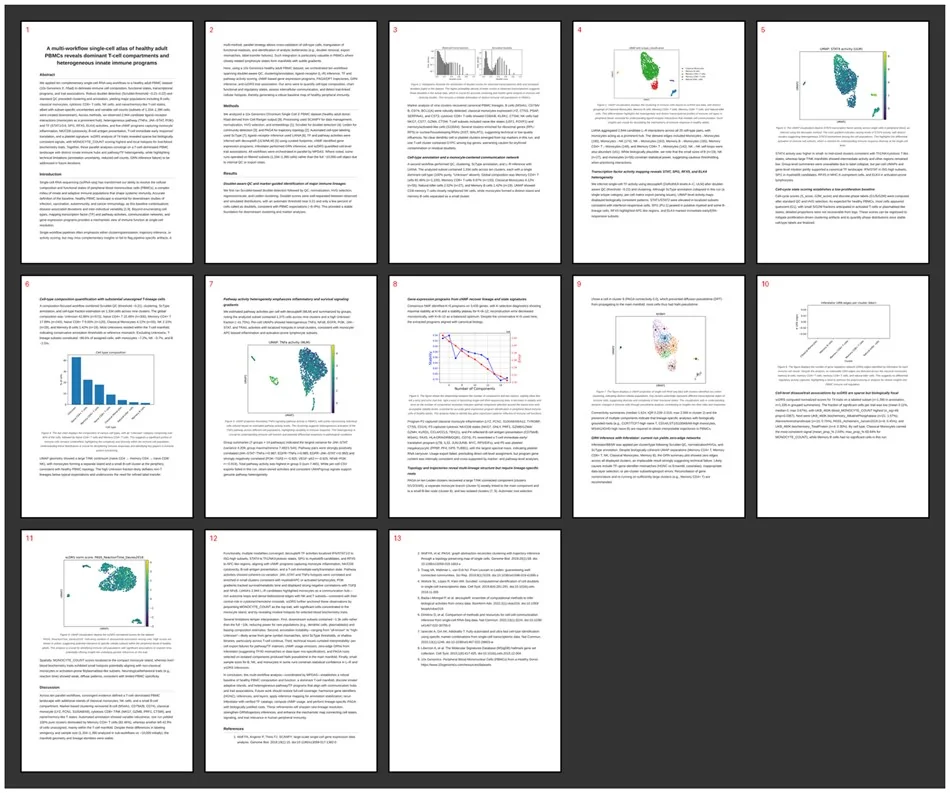
Contact sheet of the integrated interpretive report generated from the PBMC portfolio-size-10 experiment. The full report is provided as Supplementary Data S1.

## 4 Discussion

We proposed a scRNA-seq analysis framework integrating LLM-based analytical planning with workflow languages. Evaluation showed that: (i) the Validator consistently improved the executable analysis intent fulfillment rate regardless of dataset or model (overall mean 0.061→0.378); (ii) diversity of analytical themes is maintained even with a small portfolio, while the diversity of execution paths expands with portfolio size; (iii) parallel execution makes large-scale exploration practical; (iv) explicit workflow planning with structural constraints improved completion rates over ReAct (93% versus 82%) and reduced execution time approximately two-fold; (v) portfolio-level execution management—including auditability, output structure consistency, and scalability—is difficult to achieve within the sequential agent paradigm, even when equipped with the same tool suite; and (vi) automated generation of integrated interpretive reports from multiple workflow outputs is achievable.

This approach is conceptually related to multiverse analysis (Steegen *et al.* 2016) and highly comparative feature extraction (Fulcher *et al.* 2013, Fulcher and Jones 2017), but its application to scRNA-seq workflow automation is novel. Existing LLM agents such as Biomni (Huang *et al.* 2026) and CellAgent (Xiao *et al.* 2026) excel at flexibly executing user-specified strategies through code generation and tool execution. CellVoyager (Alber *et al.* 2026) goes further, generating the next hypothesis-driven analytical strategy based on existing results. However, none of these systems directly addresses the problem of explicitly constructing a portfolio of multiple independent analytical strategies in advance, compiling them into reproducible workflows after validation, and executing them in parallel.

The importance of the validator extends beyond scRNA-seq analysis. LARA-HPC (Dawson *et al.* 2026), an LLM agent framework for atomistic simulations, advocates a “paradigm shift from generation-first to validation-first” and demonstrates that validation-driven generation substantially improves the robustness of scientific workflows. Our results are consistent with this finding: the fact that technically executable workflows can fail to fulfill the intent of the analytical strategy supports the indispensability of a validation step in LLM agent–based scientific workflow generation, regardless of domain. Notably, the magnitude of improvement varied considerably by model: GPT-4o showed a large improvement (mean across three datasets: 0.017→0.600), while GPT-5 showed a smaller improvement (0.000→0.117). This demonstrates that workflow planning quality depends on the LLM used, indicating that model selection is an important factor governing practical executable analysis intent fulfillment rates.

The comparisons with ReAct and Biomni reveal that pre-execution planning has concrete advantages for both robustness and scalability. Because ReAct (Yao *et al.* 2023) determines tool-execution order step by step during inference, its execution path is vulnerable to stochastic variation in sequential LLM inference. The failure pattern observed in the cell–cell interaction analysis task—where success and failure were mixed across runs using the identical analytical strategy and model—illustrates this directly, and cannot be eliminated by reformulating the natural-language instruction alone. A complementary comparison with Biomni (Huang *et al.* 2026), connected to the same MCP servers, further demonstrates that the advantages of pre-execution planning (auditability, output structure consistency, and parallel scalability) are not readily achievable through the sequential agent paradigm alone, regardless of task completion rate.

The independence of thematic diversity from portfolio size implies that coverage of analytical themes is governed by the design of the analytical strategy generator rather than portfolio size, enabling resource-efficient multi-perspective exploration even with a small portfolio. In contrast, the diversity of execution paths expands with portfolio size, so increasing portfolio size is effective when broader exploration of the analytical space is desired. Expanding the MCP server tool catalog further broadens the analytical space itself, and the addition of new analytical methods directly expands the range of explorable strategies. Although this study adopted Snakemake, the architecture is not language-dependent and replacement with workflow languages such as Nextflow is in principle feasible.

Automated generation of integrated interpretive reports demonstrates that the framework supports not only execution of analyses but also interpretation of results. The generated reports integrate perspectives spanning cell-type identification, pseudotime trajectories, cell–cell interactions, and gene regulatory networks, enabling an overview of multi-perspective biological insights that cannot be obtained from a single pipeline. However, improving the biological depth and reliability of the final integration remains a future challenge.

Limitations include plan failure rates that depend on portfolio size and model capability, evaluation restricted to three scRNA-seq datasets (generalization to spatial transcriptomics or multiomics is unverified), and integrated report generation positioned as an initial demonstration. Future directions include extending the tool suite to additional data modalities and developing more rigorous benchmarks for biological completeness.

## 5 Conclusion

We presented a framework bridging LLM-based agents and workflow languages to automate multi-perspective scRNA-seq analysis. Plan validation substantially improved analytical correctness; diverse strategies were generated efficiently; parallelization of both plan generation and workflow execution achieved up to a 29.9-fold speedup in workflow execution, with plan generation completing in near-constant time regardless of portfolio size; and integrated reports were automatically produced. This framework provides a scalable and reproducible basis for systematic multi-perspective exploration of scRNA-seq data.

## Supplementary Material

vbag301_Supplementary_Data

## Data Availability

The datasets used in this study are publicly available: PBMC (10x Genomics 2020), mouse brain tissue (10x Genomics 2024), and human pancreatic islets (Baron *et al.* 2016).
