# Supplementary material for: Bridging large language model-based agents and workflow languages for automated multi-perspective analysis of single-cell RNA sequencing data
Source: Bioinform Adv. 2026 Oct 8;6(1):vbag301. doi: 10.1093/bioadv/vbag301 (PMC13730267; doi:10.1093/bioadv/vbag301)
Supplement: Supplementary file 1 [file vbag301_supplementary_data.pdf]

A multi-workflow single-cell atlas of healthy adult PBMCs reveals dominant T-cell compartments and heterogeneous innate immune programs

Abstract

We applied ten complementary single-cell RNA-seq workflows to a healthy adult PBMC dataset (10x Genomics 3', h5ad) to delineate immune cell composition, functional states, transcriptional programs, and trait associations. Robust doublet detection (Scrublet threshold ~0.21–0.22) and standard QC preceded clustering and annotation, yielding major populations including B cells, classical monocytes, cytotoxic CD8+ T cells, NK cells, and naive/memory-like T-cell states, albeit with subset-specific uncertainties and variable cell counts (subsets of 1,334–1,390 cells were created downstream). Across methods, we observed 2,944 candidate ligand–receptor interactions (monocytes as a prominent hub), heterogeneous pathway (TNFα, JAK–STAT, PI3K) and TF (STAT1/2/4, SPI1, RFX5, ELK4) activities, and five cNMF programs capturing monocyte inflammation, NK/CD8 cytotoxicity, B-cell antigen presentation, T-cell immediate early response/translation, and a platelet signature. scDRS analysis of 74 traits revealed sparse but biologically consistent signals, with MONOCYTE_COUNT scoring highest and local hotspots for liver/blood biochemistry traits. Together, these parallel analyses converge on a T-cell–dominated PBMC landscape with distinct innate immune hubs and pathway/TF heterogeneity, while highlighting technical limitations (annotation uncertainty, reduced cell counts, GRN inference failure) to be addressed in future iterations.

Introduction

Single-cell RNA sequencing (scRNA-seq) has transformed our ability to resolve the cellular composition and functional states of peripheral blood mononuclear cells (PBMCs), a complex milieu of innate and adaptive immune populations that shape systemic immunity. Accurate definition of the baseline, healthy PBMC landscape is essential for downstream studies of infection, vaccination, autoimmunity, and cancer immunology, as this baseline contextualizes disease-associated deviations and inter-individual variability [1,9]. Beyond enumerating cell types, mapping transcription factor (TF) and pathway activities, communication networks, and gene expression programs provides a mechanistic view of immune function at single-cell resolution.

Single-workflow pipelines often emphasize either clustering/annotation, trajectory inference, or activity scoring, but may miss complementary insights or fail to flag pipeline-specific artifacts. A

multi-method, parallel strategy allows cross-validation of cell-type calls, triangulation of functional readouts, and identification of analytic bottlenecks (e.g., doublet removal, export mismatches, label-transfer failures). Such integration is particularly valuable in PBMCs where closely related lymphocyte states form manifolds with subtle gradients.

Here, using a 10x Genomics healthy adult PBMC dataset, we orchestrated ten workflows spanning doublet-aware QC, clustering/annotation, ligand–receptor (L–R) inference, TF and pathway activity scoring, cNMF-based gene expression programs, PAGA/DPT trajectories, GRN inference, and scDRS trait association. Our aims were to quantify cell-type composition, chart functional and regulatory states, assess intercellular communication, and detect trait-linked cellular hotspots, thereby generating a robust baseline map of healthy peripheral immunity.

Methods

We analyzed a 10x Genomics Chromium Single Cell 3’ PBMC dataset (healthy adult donor; h5ad derived from Cell Ranger output) [9]. Processing used SCANPY for data management, normalization, HVG selection, and embeddings [1]; Scrublet for doublet detection [4]; Leiden for community detection [3]; and PAGA for trajectory topology [2]. Automated cell-type labeling used ScType [7]; ligand–receptor inference used LIANA [6]; TF and pathway activities were inferred with decoupleR (ULM/MLM) [5] using curated footprints; cNMF identified gene expression programs; Inferelator performed GRN inference; and scDRS quantified cell-level trait associations. All workflows were orchestrated in parallel by MPDAS. Where noted, some runs operated on filtered subsets (1,334–1,390 cells) rather than the full ~10,000-cell object due to internal QC or export steps.

Results

Doublet-aware QC and marker-guided identification of major immune lineages

We first ran Scrublet-based doublet detection followed by QC, normalization, HVG selection, regression/scale, and Leiden clustering. Doublet scores were well-separated between observed and simulated distributions, with an automatic threshold near 0.21 and only a few percent of cells called as doublets, consistent with PBMC expectations (~6–8%). This provided a stable foundation for downstream clustering and marker analyses.

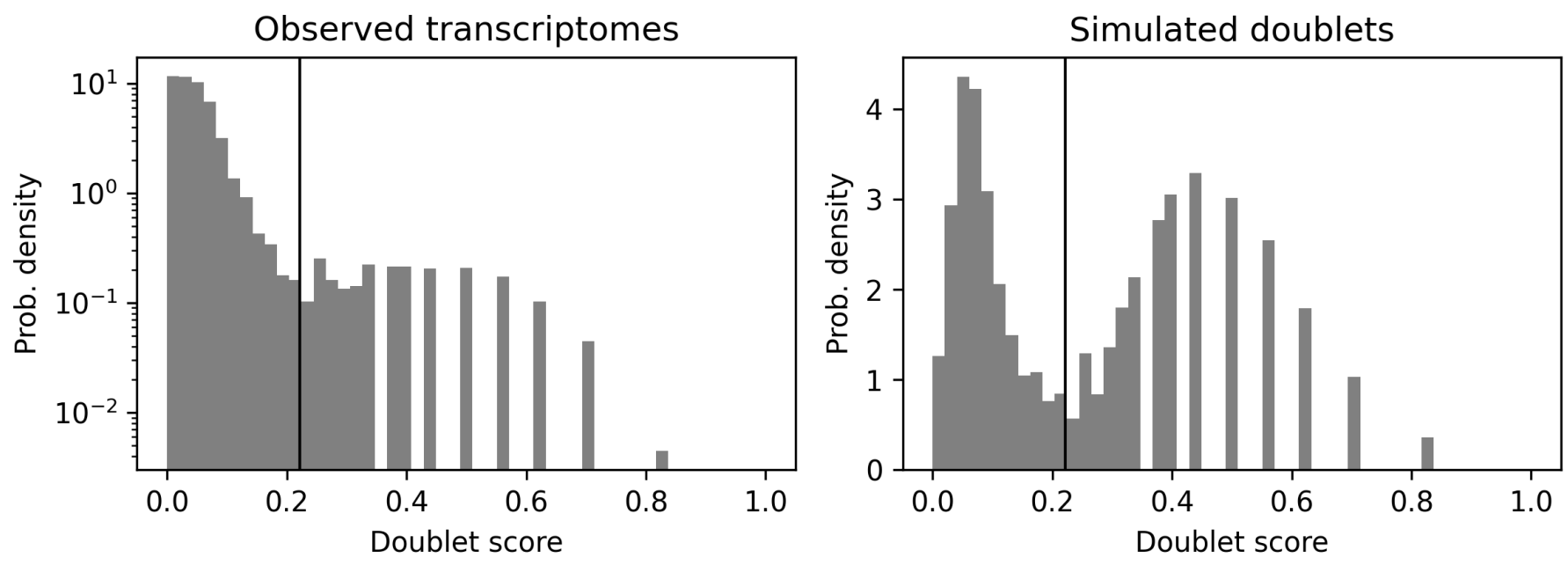

Figure 1. Histograms illustrate the distribution of doublet scores for observed transcriptomes (left) and simulated doublets (right) in the dataset. The higher probability density of lower scores in observed transcriptomes suggests fewer doublets in the actual data, which is crucial for accurate clustering and marker gene analysis in immune cell diversity studies. This ensures a reliable delineation of distinct immune cell populations in PBMCs.

Marker analysis of nine clusters recovered canonical PBMC lineages. B cells (MS4A1, CD79A/B, CD74, BCL11A) were robustly detected; classical monocytes expressed LYZ, CTSS, PSAP, SERPINA1, and CST3; cytotoxic CD8+ T cells showed CD8A/B, KLRK1, CTSW; NK cells had NKG7, CST7, GZMA, CTSW. T-cell subsets included naive-like states (LEF1, FOXP1) and memory/activated-like cells (S100A4). Several clusters enriched for ribosomal genes (RPL/RPS) or nuclear/housekeeping RNAs (XIST, MALAT1), suggesting technical or low-quality influences. No clear dendritic cell or platelet clusters emerged from top markers in this run, and one T-cell cluster contained GYPC among top genes, warranting caution for erythroid contamination or residual doublets.

Cell-type annotation and a monocyte-centered communication network

A second workflow performed QC, clustering, ScType annotation, and L–R inference with LIANA. The analyzed subset contained 1,334 cells across ten clusters, each with a single dominant cell type (100% purity, “Unknown” absent). Global composition was Memory CD4+ T cells 82.46% (n=1,100), Memory CD8+ T cells 9.97% (n=133), Classical Monocytes 4.12% (n=55), Natural killer cells 2.02% (n=27), and Memory B cells 1.42% (n=19). UMAP showed CD8 memory T cells closely neighbored NK cells, while monocytes formed a distinct island and memory B cells separated as a small cluster.

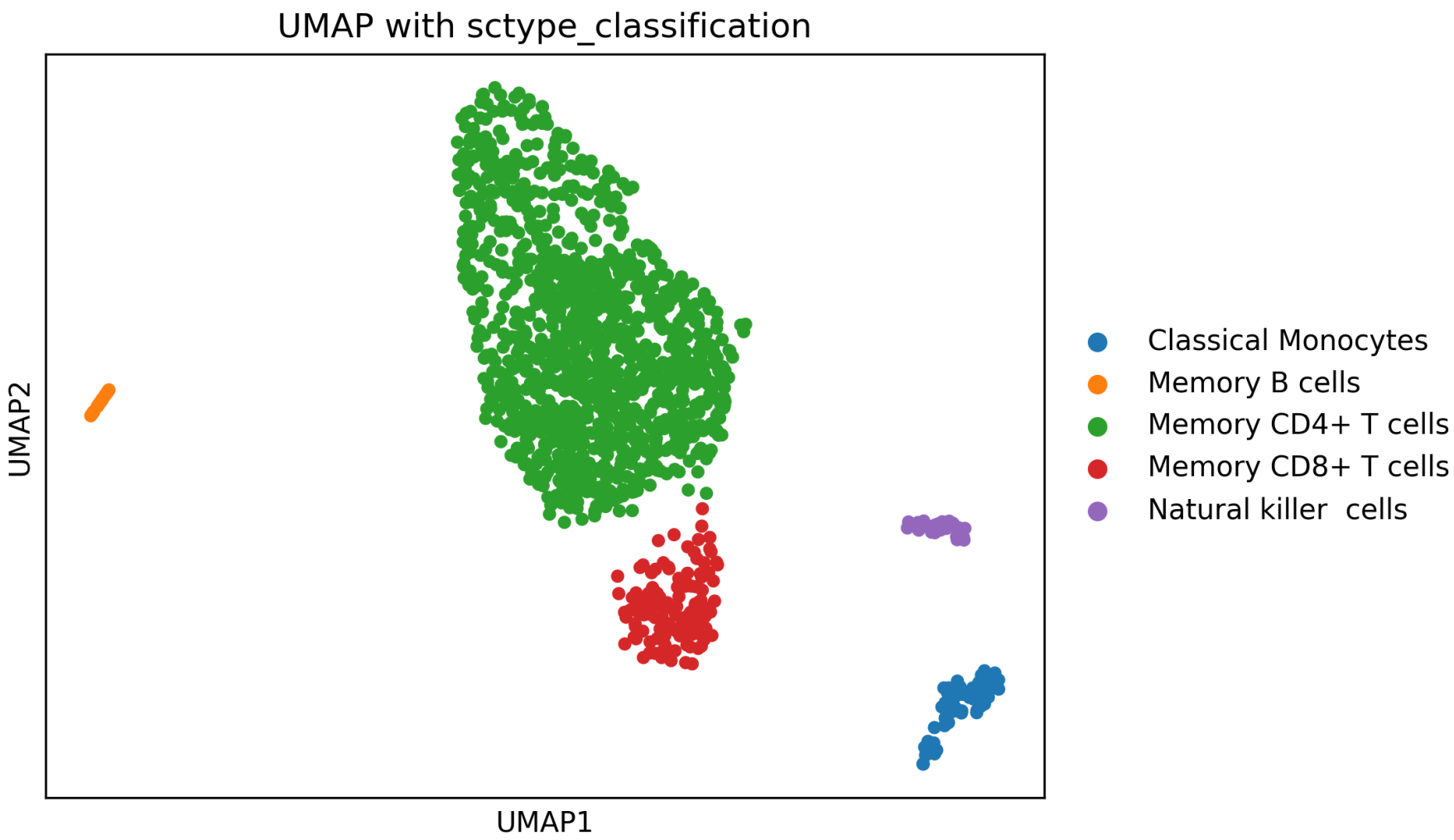

Figure 2. UMAP visualization displays the clustering of immune cells based on scRNA-seq data, with distinct groupings of Classical Monocytes, Memory B cells, Memory CD4+ T cells, Memory CD8+ T cells, and Natural killer cells. This differentiation highlights the heterogeneity and distinct transcriptional profiles of immune cell types in peripheral blood, essential for understanding ligand-receptor interactions that mediate cell communication. Such insights are crucial for elucidating the mechanisms of immune response in healthy adults.

LIANA aggregated 2,944 candidate L–R interactions across all 25 cell-type pairs, with monocytes acting as a prominent hub. The densest edges included Monocytes→Monocytes (195), Monocytes→NK (174), NK→Monocytes (163), Memory B→Monocytes (165), Memory CD4+ T→Monocytes (149), and Memory CD8+ T→Monocytes (142). NK→NK self-loops were also abundant (161). While biologically plausible, we note that the small sizes of B (n=19), NK (n=27), and monocytes (n=55) constrain statistical power, suggesting cautious thresholding when prioritizing interactions.

Transcription factor activity mapping reveals STAT, SPI1, RFX5, and ELK4 heterogeneity

We inferred single-cell TF activity using decoupleR (DoRothEA levels A–C, ULM) after doublet-aware QC (threshold ~0.22) and clustering. Although ScType annotation collapsed in this run (a single sctype category; per-cell matrix export parsing issues), UMAP-level activity maps displayed biologically consistent patterns. STAT1/STAT2 were elevated in localized subsets consistent with interferon-responsive cells, SPI1 (PU.1) peaked in putative myeloid and some B-lineage cells, RFX5 highlighted APC-like regions, and ELK4 marked immediate-early/ERK-responsive subsets.

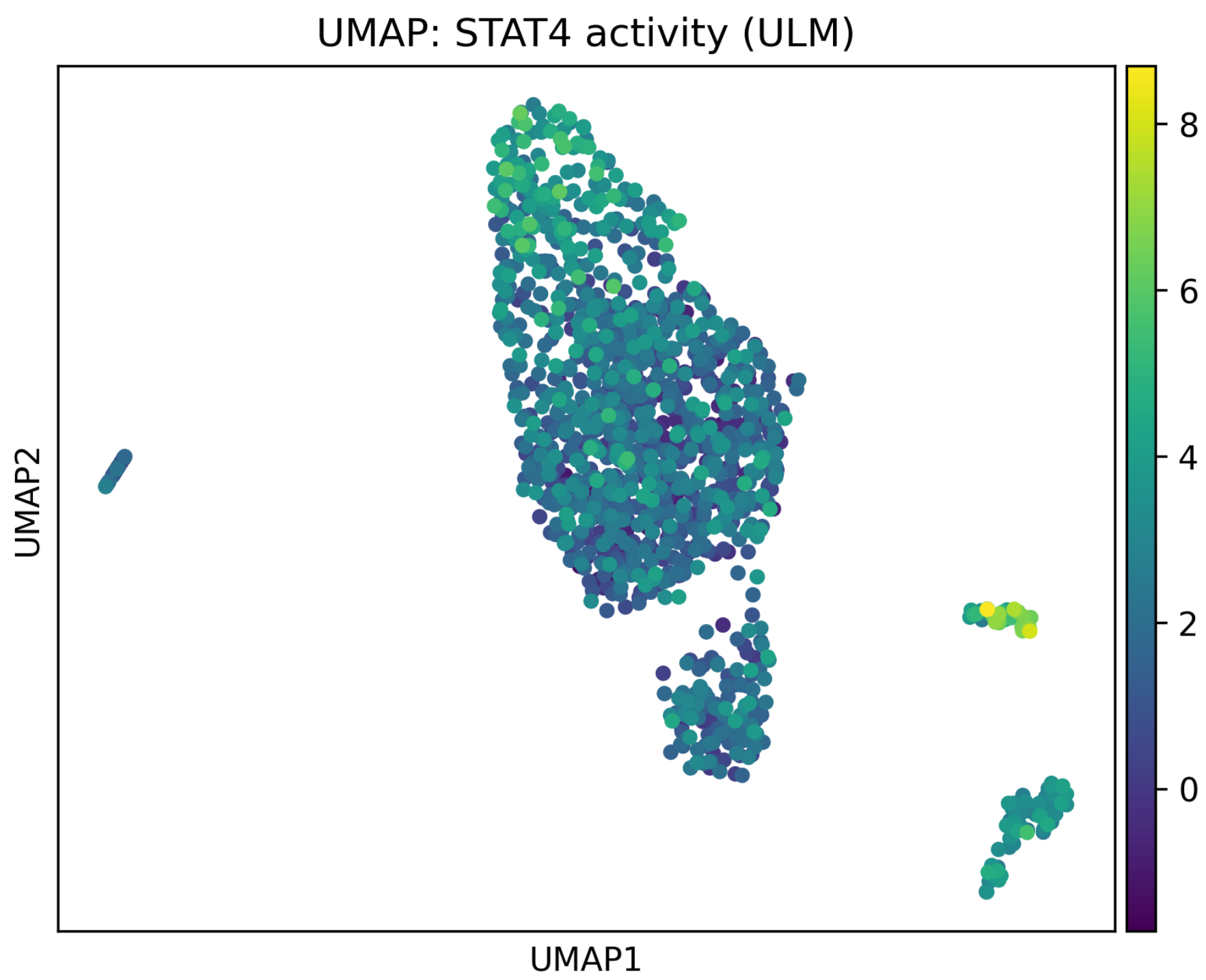

Figure 3. The UMAP visualization depicts STAT4 transcription factor activity across single cells in peripheral blood, as inferred using the decoupler method. The color gradient indicates varying levels of STAT4 activity, with distinct clusters suggesting heterogeneous STAT4 involvement among the cell populations. This highlights the differential activation of immune cell subsets, which is relevant for understanding immune response diversity at the single-cell level.

STAT4 activity was higher in small- to mid-sized clusters consistent with Th1/NK/cytotoxic T-like states, whereas large T/NK manifolds showed intermediate activity and other regions remained low. Group-level summaries were unavailable due to label collapse, but per-cell UMAPs and gene-level intuition jointly supported a canonical TF landscape: IFN/STAT in ISG-high subsets, SPI1 in myeloid/B candidates, RFX5 in MHC-II–competent cells, and ELK4 in activation-prone lymphocytes.

Cell-cycle state scoring establishes a low-proliferation baseline

Cell-cycle scores (S_score, G2M_score) and discrete phase labels (G1/S/G2M) were computed after standard QC and HVG selection. As expected for healthy PBMCs, most cells appeared quiescent (G1), with small S/G2M fractions anticipated in activated T cells or plasmablast-like states; detailed proportions were not recoverable from logs. These scores can be regressed to mitigate proliferation-driven clustering artifacts and to quantify phase distributions once stable cell-type labels are finalized.

Cell-type composition quantification with substantial unassigned T-lineage cells

A composition-focused workflow combined Scrublet QC (threshold ~0.21), clustering, ScType annotation, and cell-type fraction estimation on 1,334 cells across nine clusters. The global composition was: Unknown 42.88% (n=572), Naive CD4+ T 22.49% (n=300), Memory CD4+ T 17.99% (n=240), Naive CD8+ T 9.00% (n=120), Classical Monocytes 4.12% (n=55), NK 2.10% (n=28), and Memory B cells 1.42% (n=19). Most Unknowns resided within the T-cell manifold, indicating conservative annotation thresholds or reference mismatch. Excluding Unknowns, T-lineage subsets constituted ~86.6% of assigned cells, with monocytes ~7.2%, NK ~3.7%, and B ~2.5%.

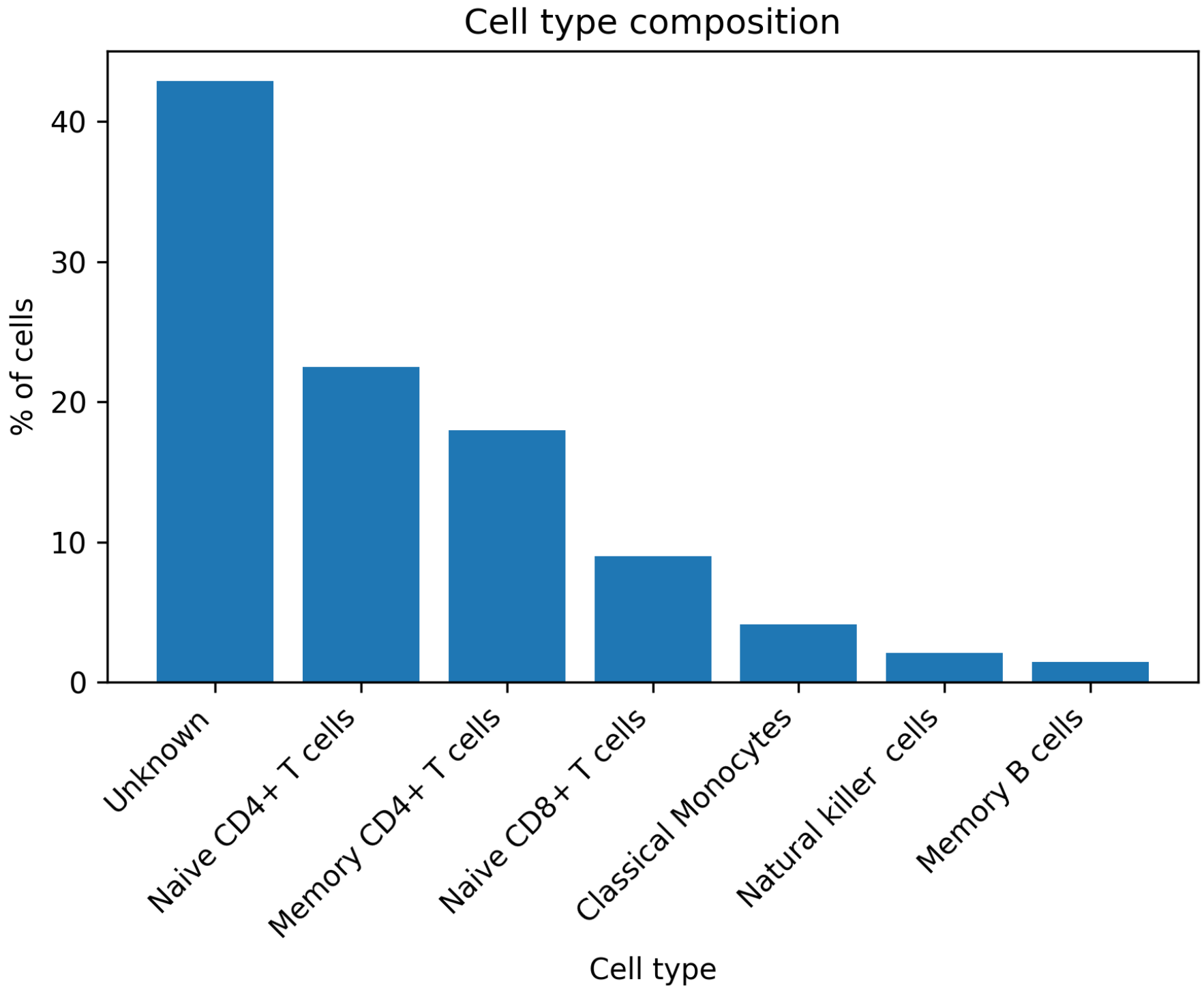

Figure 4. The bar chart displays the composition of various cell types, with an "Unknown" category comprising over 40% of the cells, followed by Naive CD4+ T cells and Memory CD4+ T cells. This suggests a significant portion of immune cells remains unidentified, highlighting the complexity and diversity within the immune cell population. Understanding these distributions is crucial for deciphering immune responses and identifying key players in immune system functioning.

UMAP geometry showed a large T/NK continuum (naive CD4 → memory CD4 → naive CD8/NK), with monocytes forming a separate island and a small B-cell cluster at the periphery, consistent with healthy PBMC topology. The high Unknown fraction likely deflates non-T lineages below typical expectations and underscores the need for refined label transfer.

Pathway activity heterogeneity emphasizes inflammatory and survival signaling gradients

We estimated pathway activities per cell with decoupleR (MLM) and summarized by groups, noting the analyzed subset contained 1,370 cells across nine clusters and a high Unknown fraction (~41.75%). Per-cell UMAPs showed heterogeneous TNFα, NFκB, EGFR, PI3K, JAK–STAT, and TRAIL activities with localized hotspots in small clusters, consistent with monocyte/APC-biased inflammation and activation-prone lymphocyte subsets.

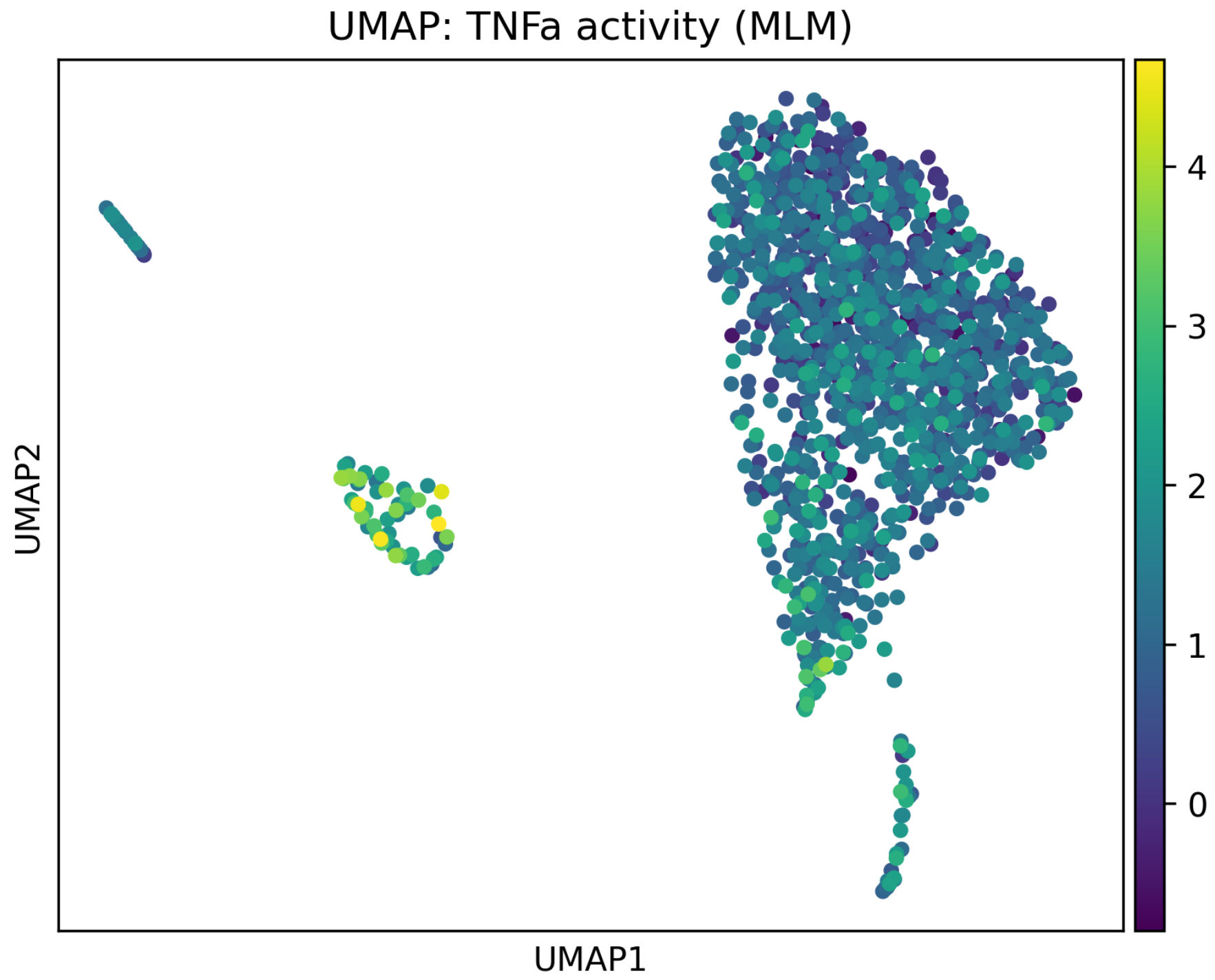

Figure 5. UMAP projection illustrating TNFα signaling pathway activity in PBMCs, with points representing individual cells colored based on estimated pathway activity levels. The clustering suggests heterogeneous activation of the TNFα pathway across different cell populations, highlighting variability in immune response. This heterogeneity is crucial for understanding immune cell function and potential differential responses in pathological conditions.

Group summaries (7 groups × 14 pathways) indicated the largest variance for JAK–STAT (variance 4.209; group maxima/minima 7.482/1.544). Pathway pairs were strongly positively correlated (JAK–STAT~TNFα r=0.987; EGFR~TNFα r=0.985; EGFR~JAK–STAT r=0.950) and strongly negatively correlated (PI3K~TGFβ r=−0.925; VEGF~p53 r=−0.925; NFκB~PI3K r=−0.919). Total pathway activity was highest in group 0 (sum 7.465). While per-cell CSV exports failed in this run, obsm-stored activities and consistent UMAP/group signals support genuine pathway heterogeneity.

Gene expression programs from cNMF recover lineage and state signatures

Consensus NMF identified K=5 programs on 3,435 genes, with K-selection diagnostics showing maximal stability at K=6 and a stability plateau for K=8–12; reconstruction error decreased monotonically, with K≈9–10 as a balanced optimum. Despite the conservative K=5 used here, the extracted programs aligned with canonical biology.

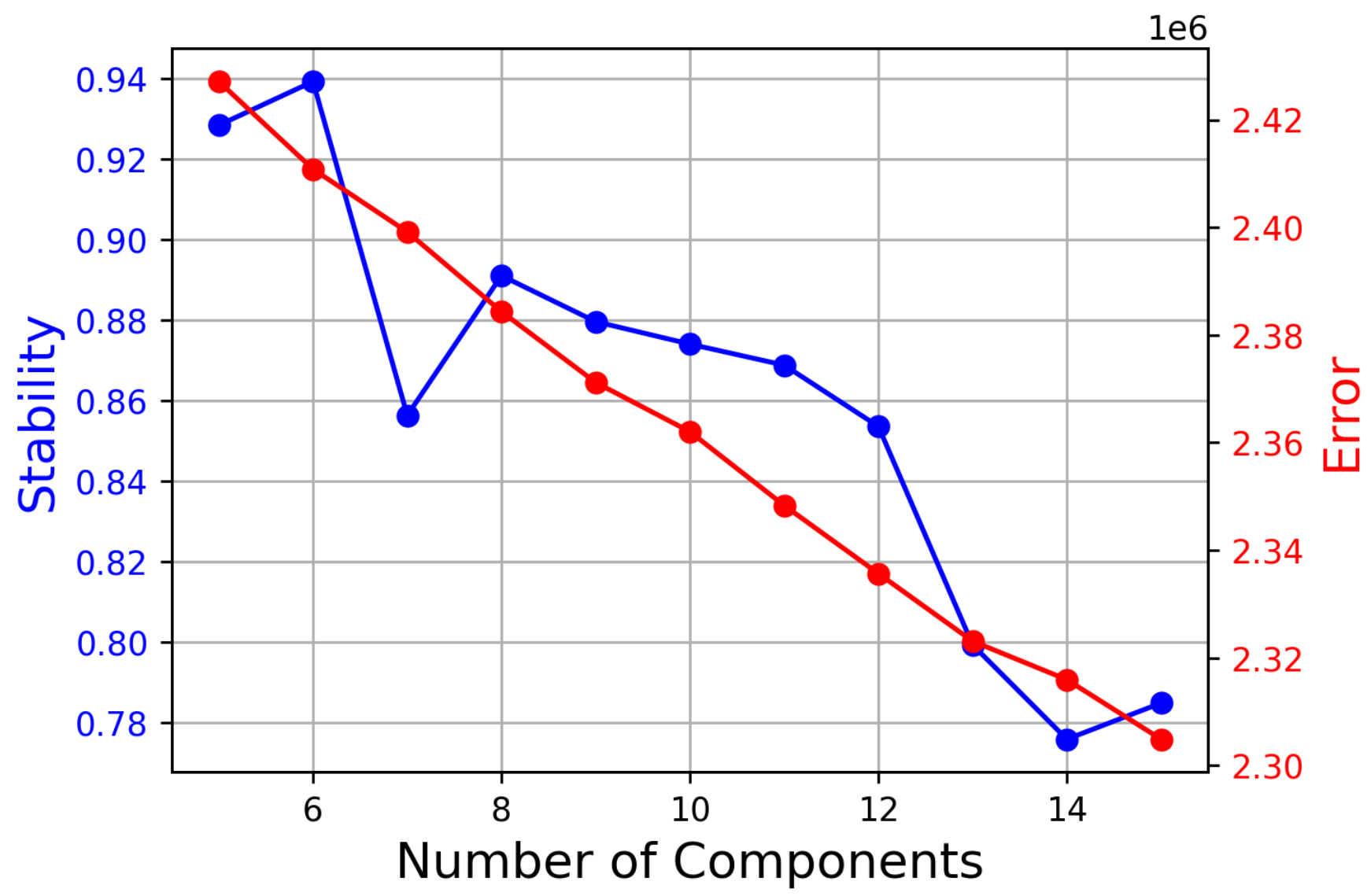

Figure 6. The figure shows the relationship between the number of components and two metrics: stability (blue line, left y-axis) and error (red line, right y-axis) in factorizing single-cell RNA sequencing data. A decrease in stability and error as the number of components increases indicates optimal component selection around the lowest error and acceptable stability levels, essential for accurate gene expression program identification in peripheral blood immune cells of healthy adults. This analysis helps to identify key gene expression patterns reflective of immune cell functions.

Program P2 captured classical monocyte inflammation (LYZ, FCN1, S100A8/A9/A12, TYROBP, CTSS, CD14), P3 captured cytotoxic NK/CD8 states (NKG7, GNLY, PRF1, GZMB/GZMA/GZMH, KLRD1, CCL4/CCL5, TBX21), and P4 reflected B-cell antigen presentation (CD79A/B, MS4A1, PAX5, HLA-DRA/DRB/DQB1, CD74). P1 resembled a T-cell immediate-early/translation program (LTB, IL32, JUN/JUNB, MYC, RPS/EIFs), and P5 was platelet/megakaryocytic (PPBP, PF4, GP9, TUBB1), with the largest spectral mass, indicating platelet RNA carryover. Usage export failed, precluding direct cell-level assignment, but program gene content was internally consistent and cross-supported by marker- and pathway-level analyses.

Topology and trajectories reveal multi-lineage structure but require lineage-specific roots

PAGA on ten Leiden clusters recovered a large T/NK-connected component (clusters 0/1/2/3/4/6), a separate monocyte branch (cluster 5) weakly linked to the main component and to a small B-like node (cluster 8), and two isolated clusters (7, 9). Automatic root selection

chose a cell in cluster 9 (PAGA connectivity 0.0), which prevented diffusion pseudotime (DPT) from propagating to the main manifold; most cells thus had NaN pseudotime.

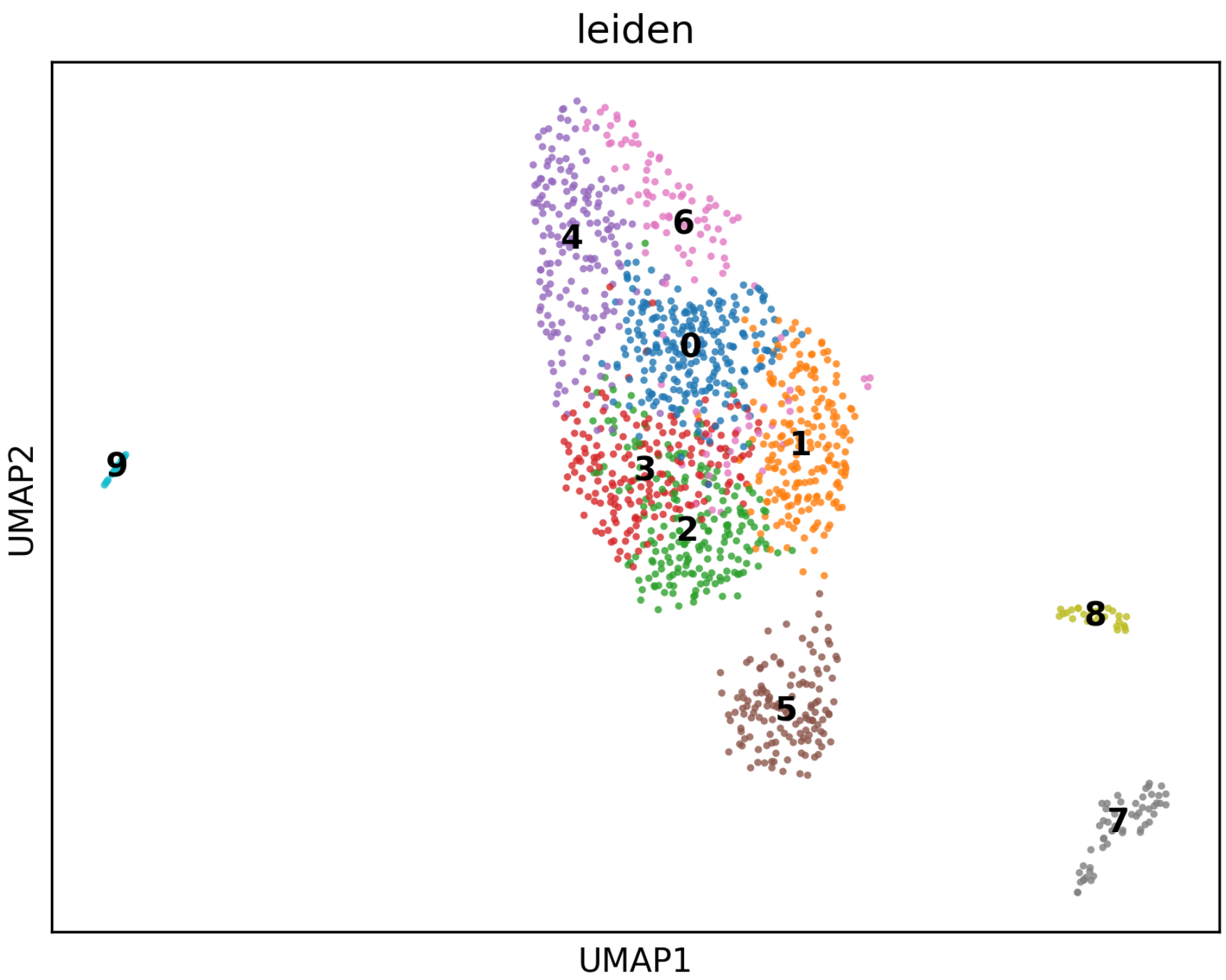

Figure 7. The figure displays a UMAP projection of single-cell RNA-seq data with clusters identified via Leiden clustering, indicating distinct cellular populations. Key clusters potentially represent different transcriptional states of immune cells, suggesting diversity and complexity in their functional states. This visualization aids in understanding dynamic changes in immune cells through pseudotime analysis, contributing to insights into their roles and responses.

Connectivity summaries (median 1.624; IQR 0.228–2.015; max 2.595 in cluster 2) and the presence of multiple components indicate that lineage-specific analyses with biologically grounded roots (e.g., CCR7/TCF7-high naive T, CD14/LST1/S100A8/A9-high monocytes, MS4A1/IGHD-high naive B) are required to obtain interpretable trajectories in PBMCs.

GRN inference with Inferelator: current run yields zero-edge networks

Inferelator/BBSR was applied per cluster/type following Scrublet QC, normalization/HVGs, and ScType annotation. Despite biologically coherent UMAP separations (Memory CD4+ T, Memory CD8+ T, NK, Classical Monocytes, Memory B), the GRN summary plot showed zero edges across all displayed clusters, an implausible result strongly suggesting technical failure. Likely causes include TF–gene identifier mismatches (HGNC vs Ensembl, case/alias), inappropriate data layer selection, or per-cluster subsetting/export errors. Reconciliation of gene nomenclature and re-running on sufficiently large clusters (e.g., Memory CD4+ T) are recommended.

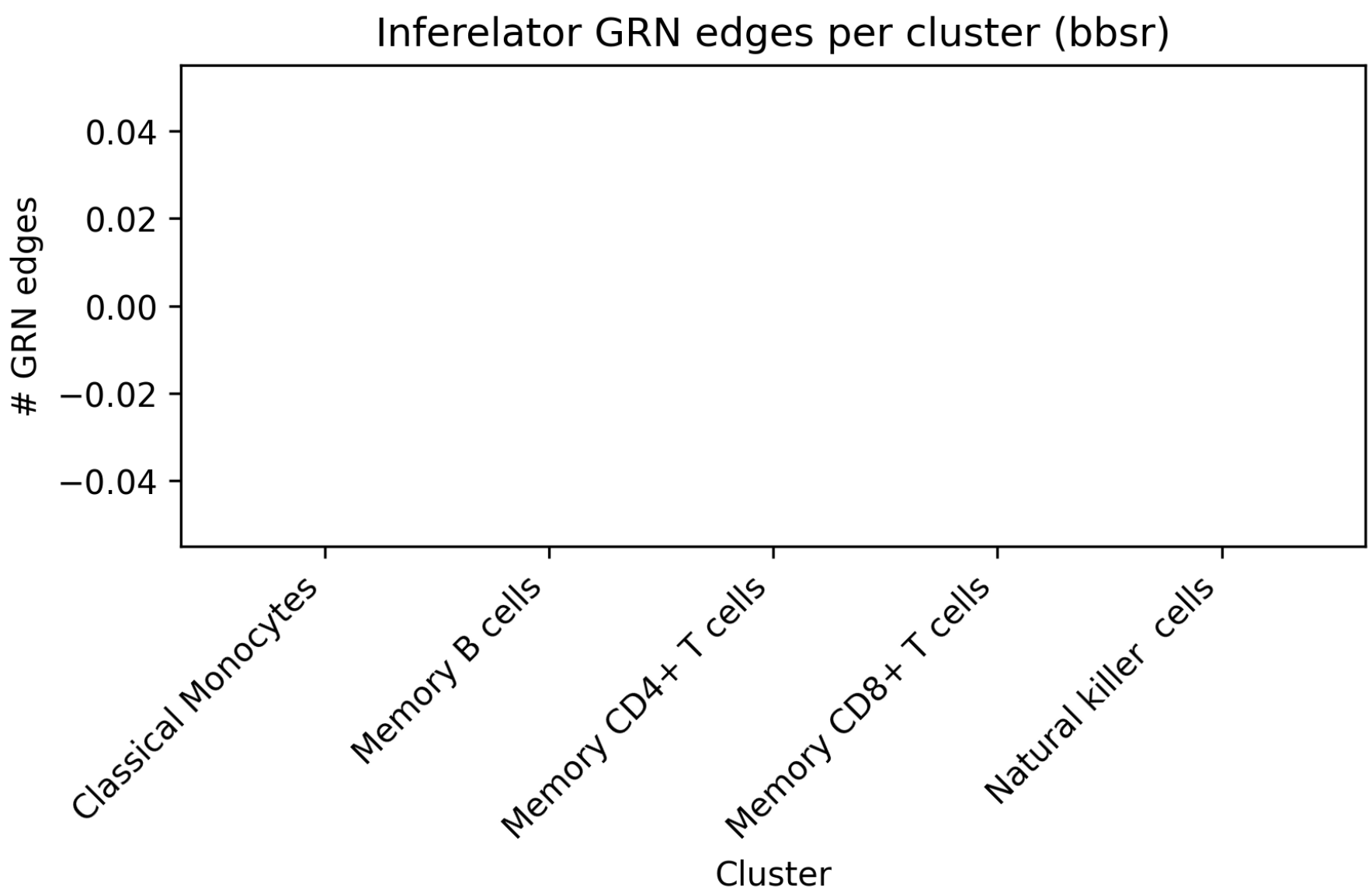

Figure 8. The figure displays the number of gene regulatory network (GRN) edges identified by Inferelator for each immune cell cluster. Despite the analysis, no noticeable GRN edges are detected across the classical monocytes, memory B cells, memory CD4+ T cells, memory CD8+ T cells, and natural killer cells. This suggests no differential regulatory activity captured, highlighting a need to optimize the preprocessing or analysis for clearer insights into PBMC immune cell regulation.

Cell-level disease/trait associations by scDRS are sparse but biologically focal

scDRS computed normalized scores for 74 traits on a labeled subset (n=1,390 in annotation; n=1,335 in grouped summaries). The fraction of significant cells per trait was low (mean 0.11%, median 0, max 3.67%), with UKB_460K.blood_MONOCYTE_COUNT highest (n_sig=49; prop=0.0367). Next were UKB_460K.biochemistry_AlkalinePhosphatase (n=21; 1.57%), AlanineAminotransferase (n=10; 0.75%), PASS_Alzheimers_Jansen2019 (n=6; 0.45%), and UKB_460K.biochemistry_TotalProtein (n=4; 0.30%). By cell type, Classical Monocytes carried the most consistent signal (mean_prop_% 2.04%; max_prop_% 83.64% for MONOCYTE_COUNT), while Memory B cells had no significant cells in this run.

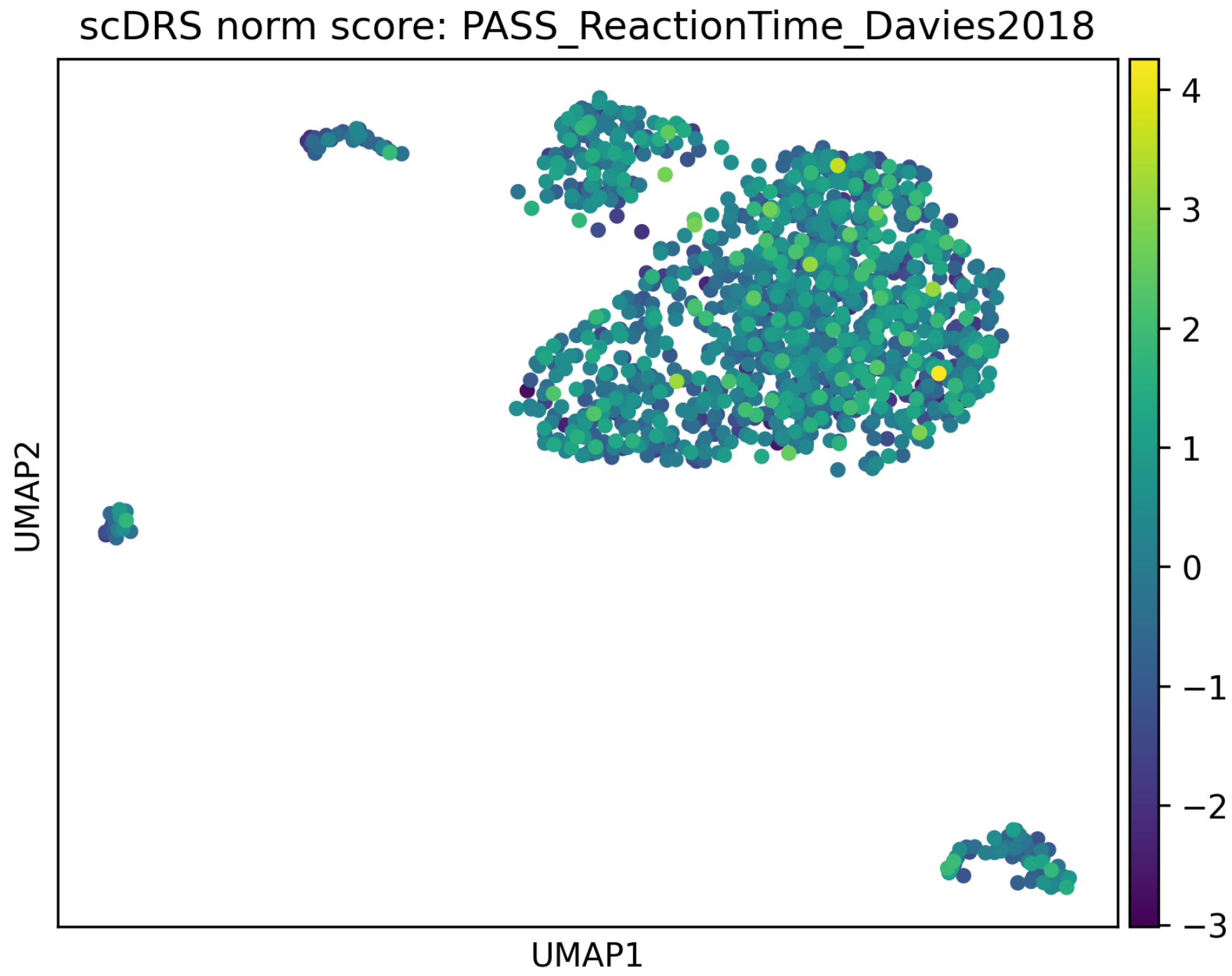

Figure 9. UMAP visualization depicts the scDRS normalized scores for the dataset "PASS_ReactionTime_Davies2018," indicating variation in disease/trait association among cells. High scores are shown in yellow, suggesting potential relevance to specific cellular subsets within the peripheral blood of healthy adults. This analysis is crucial for identifying immune cell populations with significant associations to reaction time, potentially offering insight into underlying genetic influences on this trait.

Spatially, MONOCYTE_COUNT scores localized to the compact monocyte island, whereas liver/blood biochemistry traits exhibited small hotspots potentially aligning with non-classical monocytes or activation-prone B/plasmablast-like subsets. Neurological/behavioral traits (e.g., reaction time) showed weak, diffuse patterns, consistent with limited PBMC specificity.

Discussion

Across ten parallel workflows, convergent evidence defined a T-cell–dominated PBMC landscape with additional islands of classical monocytes, NK cells, and a small B-cell compartment. Marker-based clustering recovered B-cell (MS4A1, CD79A/B, CD74), classical monocyte (LYZ, FCN1, S100A8/A9), cytotoxic CD8+ T/NK (NKG7, GZMB, PRF1, CTSW), and naive/memory-like T states. Automated annotation showed variable robustness: one run yielded 100% pure clusters dominated by Memory CD4+ T cells (82.46%), whereas another left 42.9% of cells unassigned, mainly within the T-cell manifold. Despite these differences in labeling stringency and sample size (1,334–1,390 analyzed in sub-workflows vs ~10,000 initially), the manifold geometry and lineage identities were stable.

Functionally, multiple modalities converged. decoupleR TF activities localized IFN/STAT1/2 to ISG-high subsets, STAT4 to Th1/NK/cytotoxic states, SPI1 to myeloid/B candidates, and RFX5 to APC-like regions, aligning with cNMF programs capturing monocyte inflammation, NK/CD8 cytotoxicity, B-cell antigen presentation, and a T-cell immediate-early/translation state. Pathway activities showed coherent co-variation: JAK–STAT and TNFα hotspots were correlated and enriched in small clusters consistent with myeloid/APC or activated lymphocytes; PI3K gradients tracked survival/metabolic tone and displayed strong negative correlations with TGFβ and NFκB. LIANA's 2,944 L–R candidates highlighted monocytes as a communication hub—rich autocrine loops and dense bidirectional edges with NK and T subsets—consistent with their central role in cytokine/chemokine crosstalk. scDRS further anchored these observations by pinpointing MONOCYTE_COUNT as the top trait, with significant cells concentrated in the monocyte island, and by revealing modest hotspots for selected blood biochemistry traits.

Several limitations temper interpretation. First, downstream subsets contained ~1.3k cells rather than the full ~10k, reducing power for rare populations (e.g., dendritic cells, plasmablasts) and biasing composition estimates. Second, annotation instability—ranging from "all-known" to "high-Unknown"—likely arose from gene symbol mismatches, strict ScType thresholds, or shallow libraries, particularly across T-cell continua. Third, technical issues curtailed interpretability: per-cell export failures for pathway/TF matrices, cNMF usage omission, zero-edge GRNs from Inferelator (suggesting TF/ID mismatches or data-layer mis-specification), and PAGA roots selected on isolated components produced NaN pseudotime in the main manifold. Finally, small sample sizes for B, NK, and monocytes in some runs constrain statistical confidence in L–R and scDRS inferences.

In conclusion, this multi-workflow analysis—coordinated by MPDAS—establishes a robust baseline of healthy PBMC composition and function: a dominant T-cell manifold, discrete innate/adaptive islands, and heterogeneous pathway/TF programs that align with communication hubs and trait associations. Future work should restore full-cell coverage; harmonize gene identifiers (HGNC), references, and layers; apply reference mapping for annotation stabilization; rerun Inferelator with verified TF catalogs; compute cNMF usage; and perform lineage-specific PAGA with biologically justified roots. These refinements will sharpen rare-lineage resolution, strengthen GRN/trajectory inferences, and enhance the mechanistic map connecting cell states, signaling, and trait relevance in human peripheral immunity.

References

1. Wolf FA, Angerer P, Theis FJ. SCANPY: large-scale single-cell gene expression data analysis. Genome Biol. 2018;19(1):15. doi:10.1186/s13059-017-1382-0

2. Wolf FA, et al. PAGA: graph abstraction reconciles clustering with trajectory inference through a topology preserving map of single cells. Genome Biol. 2019;20(1):59. doi: 10.1186/s13059-019-1663-x
3. Traag VA, Waltman L, van Eck NJ. From Louvain to Leiden: guaranteeing well-connected communities. Sci Rep. 2019;9(1):5233. doi:10.1038/s41598-019-41695-z
4. Wolock SL, Lopez R, Klein AM. Scrublet: computational identification of cell doublets in single-cell transcriptomic data. Cell Syst. 2019;8(4):281-291. doi:10.1016/j.cels.2018.11.005
5. Badia-i-Mompel P, et al. decoupleR: ensemble of computational methods to infer biological activities from omics data. Bioinform Adv. 2022;2(1):vbac016. doi:10.1093/bioadv/vbac016
6. Dimitrov D, et al. Comparison of methods and resources for cell-cell communication inference from single-cell RNA-Seq data. Nat Commun. 2022;13(1):3224. doi:10.1038/s41467-022-30755-0
7. Ianevski A, Giri AK, Aittokallio T. Fully-automated and ultra-fast cell-type identification using specific marker combinations from single-cell transcriptomic data. Nat Commun. 2022;13(1):1246. doi:10.1038/s41467-022-28803-w
8. Liberzon A, et al. The Molecular Signatures Database (MSigDB) hallmark gene set collection. Cell Syst. 2015;1(6):417-425. doi:10.1016/j.cels.2015.12.004
9. 10x Genomics. Peripheral Blood Mononuclear Cells (PBMCs) from a Healthy Donor. https://www.10xgenomics.com/resources/datasets
